# The Impact of Stand-Biased Desks on Afterschool Physical Activity Behaviors of Elementary School Children

**DOI:** 10.3390/ijerph19137689

**Published:** 2022-06-23

**Authors:** Nathan R. Tokarek, Chi C. Cho, Scott J. Strath, Ann M. Swartz

**Affiliations:** 1 Department of Kinesiology, College of Health Sciences, University of Wisconsin, Milwaukee, WI 53211, USA; sstrath@uwm.edu (S.J.S.); aswartz@uwm.edu (A.M.S.); 2 Physical Activity and Health Research Lab, University of Wisconsin, Milwaukee, WI 53211, USA; 3 Center for Aging and Translational Research, University of Wisconsin, Milwaukee, WI 53211, USA; chocc@uwm.edu

**Keywords:** student, environment, sedentary, sit, intervention, free living, movement

## Abstract

The purpose of this secondary analysis was to assess whether students’ use of stand-biased desks during the school day influenced physical activity (PA) and sedentary behaviors (SB) during the afterschool period. By using a crossover design consisting of two 9-week intervention periods, 99 participants from grades 3, 4, and 6 were randomly assigned by their teacher to either a traditional (Group 1; sit–stand) or stand-biased (Group 2; stand–sit) desk in the classroom. The desk type then switched between intervention periods. Afterschool PA and SB were measured by accelerometry at baseline (fall) and following both intervention periods at post I (winter) and post II (spring). Independent sample *t*-tests and mixed-effects modeling were applied at a significance value of *p* < 0.05 to detect differences between groups. No significant differences in afterschool SB, light-intensity PA (LPA), or moderate- to vigorous-intensity PA (MVPA) were found between groups. There were also no significant two- or three-way interaction effects detected between desk assignment, time, and afterschool SB, LPA, or MVPA. Stand-biased desks in the classroom were not detrimental to children’s afterschool PA and SB.

## 1. Introduction

Children currently spend upwards of 8 h per day, or an average of 62% of all waking hours engaged in sedentary behaviors (SB) [1]. Consequently, only 24% of children between 6 and 17 years of age are meeting the physical activity (PA) recommendation of 60-min of moderate- to vigorous-intensity PA (MVPA) on all days of the week [2], and the prevalence of meeting these recommendations decreases with age. In children, increased levels of SB have been linked to an increased risk of developing obesity [3], decreased aerobic fitness [4], and an increase in metabolic risk factors [5,6]. Additionally, high levels of SB have a negative impact on children’s psychological well-being, including reductions in self-esteem, academic achievement, and increases in symptoms of depression [7,8,9,10]. Meanwhile, children and adolescents who meet or exceed PA recommendations experience weight loss and maintenance, the strengthening of muscle and bone, and a reduced risk of chronic disease [11], as well as improvements in cognitive ability [12] and attitude [11]. In this sense, PA and SB independently impact a child’s health, in that insufficient PA and excess SB each carry negative health risks capable of compounding one another [13]. Therefore, efforts to reduce children’s SB and increase PA may help to improve the current and future health of today’s children.

More than 56 million students within the United States attend a public or private school on a regular basis [14,15]. During the school year, a large proportion of a child’s waking day is spent in school, and it has been suggested that children spend more than 75% of an average 6-h school day sitting [16]. At the same time, educators are encouraged to provide students with opportunities throughout the in-school period to accumulate a minimum of 30 min of MVPA to aid in meeting PA recommendations during the school week [17]. Therefore, the school setting presents an ideal opportunity in which the PA and SB levels of a large proportion of children living in the United States can be influenced [18]. When considering the implementation of school-based interventions to alter PA and SB, however, it is crucial that researchers consider the potential impact on activity behaviors across the entire day. It has previously been shown that children may compensate for in-school changes in PA and SB by increasing or decreasing activity levels at a later time, either in the same day or in the days following [19,20]. Therefore, when targeting a reduction in children’s in-school SB through a more active school day, the most effective school-based interventions should ideally not result in increased sedentary behavior after school.

Stand-biased desks, as the name suggests, are desks which can be adjusted to comfortably support an individual to do work while in a standing position, or while seated on a height-appropriate stool. Stand-biased desks in the classroom provide a novel approach to modifying children’s sitting time during the school day to incorporate more standing, while theoretically having a minimal impact on children’s learning environment. To date, stand-biased desk interventions have primarily focused on the impacts which occur across the school day. Among these, researchers have reported positive findings in sitting and standing behaviors [21,22,23,24,25,26], energy expenditure while standing in the classroom [27,28,29], postural improvements during use [30], and increases in cognitive function, particularly in the realms of working memory, executive function, and academic engagement [31,32].

Following the implementation of stand-biased desks into the classroom to directly influence in-school PA and SB, it remains to be examined whether student’s activity behaviors during other periods of the day, such as after school, are impacted.

The purpose of this secondary analysis is to assess whether stand-biased desk use in the classroom is associated with elementary student’s engagement in SB, light-intensity PA (LPA), and MVPA during the afterschool period. Specifically, this analysis aimed to compare the afterschool PA and SB of a sample of third-, fourth-, and sixth-grade students assigned to use either a stand-biased or traditional seated desk during the school day. It was hypothesized that the utilization of stand-biased desks in the classroom would not result in significant alterations to activity levels during the afterschool period.

## 2. Materials and Methods

### 2.1. Design

This study is a secondary analysis of data collected during the 2016–2017 academic year using a single-site, non-randomized, two-arm control trial with a crossover design [26]. Data were analyzed between September 2016 and April 2017. Baseline assessments were completed early in the fall while all students were in a seated desk. All students in the classroom (whether participating or not) were assigned seating (seated desk/control condition or stand-biased desk/intervention) by the classroom teacher. Group 1 (“sit–stand”) was assigned to a seated desk for the first nine-week period, then to a stand-biased desk for the second nine-week period. Group 2 (“stand–sit”) was assigned to a stand-biased desk for the first nine-week period, then to a seated desk for the second nine-week period. Assessments were performed at baseline (fall), at the end of the first intervention period (post I; winter), and at the end of the second intervention period (post II; spring).

### 2.2. Participants

Students from participating third-, fourth-, and sixth-grade classrooms within a single elementary school were invited to enroll in this study.

Classroom eligibility included those which encompassed an entire grade level (i.e., all grade 6 classrooms), allowing for students to maintain their desk assignment between classroom rotations throughout the school day. Inclusion criteria for students in these classrooms included: (1) the ability to read and understand the English language, and (2) no physical constraints preventing participating students from being able to stand or move without limitation.

This study was approved by the Institutional Review Board at the University of Wisconsin–Milwaukee (IRB # 17.019). Informed written consent was obtained from all primary caregivers and all participating students provided assent to participate in the study.

### 2.3. Intervention

The goal of the original study was to examine the impact of stand-biased desks on the in-school PA and SB of elementary school children. To address this aim, an environmental intervention was applied. Within each participating classroom, half of the traditional desks were replaced with a stand-biased desk. Students assigned to the stand-biased desks were encouraged to stand while at their desks. Teachers provided reminders, encouragement, and activities that would promote standing.

### 2.4. Measurements

Data was collected at three time points: baseline, following the first intervention period (post I), and following the second, crossover intervention period (post II). Data collected included the following.

Demographic Information: Upon enrollment into the study, primary caregivers were asked to complete a child and family health and demographics questionnaire for each student.

Sport Participation & Active Transportation Home from School: A single question was drawn from both a “perceptions” questionnaire administered to primary caregivers, and the Youth Activity Profile [33] administered to participating students. Surveys were administered during each assessment period, and primary caregivers provided information regarding their child’s participation in sport or organized activities after school, while students self-reported on active transport from school to home.

Physical Activity & Sedentary Behaviors: At baseline, post I, and post II, participating students wore an Actigraph accelerometer (GT3X+ or wGT3X-BT; Pensacola, FL, USA) on an elastic belt fastened around the waist, with the device placed at the anterior aspect of the right hip, according to manufacturer instructions. These devices were handed out at the beginning of the school day on the first day of each data collection week. Participants were instructed to wear the device during all waking hours, from the moment they woke up, until they went to sleep. Devices were collected on the last day of the school week at the end of the school day, prior to participants going home for the weekend. In addition to receiving verbal and written accelerometer wear instructions, participants were also provided with a wear log to record the times at which a device was put on in the morning, taken off at night, and any periods throughout the day in which participants did not wear the device (such as when showering, swimming, etc.).

Weather: Hourly afterschool weather data was sourced from National Oceanic and Atmospheric Administration (NOAA) records for the region in which the study took place. This descriptive environmental data included the variables of outdoor temperature, relative humidity, precipitation, wind speed, and time of sunset. All data were averaged across each afterschool and total data collection period.

### 2.5. Data Analysis

The accelerometers used in this study provided time-stamped data permitting the exploration of the afterschool period as a standalone component of the day. Data was downloaded by using Actilife software (Actigraph, Pensacola, FL, USA) and filtered on a day-by-day basis. Participants returned accelerometers at the end of the school day on the last day of the school week during each data collection period. During baseline and post I collection periods, a maximum of five days of in-school wear and four days of afterschool wear time was able to be analyzed per participant at each point. Post II data collection occurred during the Easter holiday period; therefore, four days of in-school and three days of after-school wear were recorded. In-school wear time began with the start of the school day, lasting until school release on each specific day of the week. The school day began at 8:15 am each day, and ended at 3:15 pm on Monday (M), Tuesday (T), and Thursday (Th); however, on Wednesday (W) school released at 2:30 pm.

For this secondary analysis, we aimed to examine the period of time between school dismissal and bed time, labeled the “afterschool period” in this study. In order to account for day-to-day variations in school release, maintain a standardized amount of time for behavior analysis, and account for the recommendation that children 6–12 years of age sleep for 9–12 h per 24-h period to promote optimal health [34], we defined the afterschool period as the six hours after school release. In total, participants had the ability to provide up to 24 h of afterschool wear time (six hours per day for four days, M-Th) across baseline and post I data collection periods, and 18 h of afterschool wear time (six hours per day for three days, T-Th) during the post II period, for a total of 66 h. Accelerometer non-wear time was assessed by using the algorithm developed by Choi et al. [35], and activity intensity cutpoints developed by Evenson et al. [36] were applied to the data to determine time spent demonstrating sedentary behavior (SB; 0–25 counts/15 s), light-intensity physical activity (LPA; 26–573 counts/15 s), and moderate- to vigorous-intensity physical activity (MVPA; ≥574 counts/15 s). Time spent in SB, LPA, and MVPA were also converted to a proportion (%) of total afterschool wear time. A valid afterschool period was defined as a minimum of 3 h and 47 min, or 63% of the possible six hours of wear time after school. This cutoff value was based on criteria for a valid wear day published by Colley and colleagues [37], suggesting that 10 of 16 waking hours (63%) provides a valid estimation of habitual PA in adults. Participants were included in the data analysis if they provided a minimum of 1 day of valid data per wear period. The number of valid wear days provided by participants during each assessment period is provided in Table 1.

### 2.6. Statistical Analysis

Participant characteristics are presented within groups as a mean ± standard deviation (SD) or as a frequency and percentage of the total sample. Participant characteristics were compared across grade levels for significant differences by using Kruskal–Wallis, chi-square, and Fisher’s exact tests. Accelerometer and survey data were compared by using independent sample *t*-tests, chi-square tests of association, trend analysis, and Wilcoxon rank sum tests to determine whether significant differences existed between desk types during each measurement period. Mixed-effects modeling was then used to estimate the effects of desk assignment, time period, and in-school levels of SB, LPA, or MVPA on the proportion of time participants spent in SB, LPA, and MVPA during the afterschool period. Statistical significance was determined by using a *p*-value < 0.05 and all statistical analysis were performed by using SAS 9.4 (Cary, NC, USA).

## 3. Results

### 3.1. Sample and School Descriptives

Students participating in this study were from a single elementary school located in a large, Midwestern metropolitan area in the United States. A total of 99 students from third (*n* = 22), fourth (*n* = 36), and sixth (*n* = 41) grade enrolled in this study. Of those who provided complete demographic information, participating students were predominately male (57.1%), white (79.3%), and from households reporting an annual income of $100,000 or more (62.8%). Across all three grades, participants also recorded an average BMI age- and sex-matched percentile of 52.6 ± 29.9%. Participant characteristics by grade level can be found in Table 2.

Age, height, body mass, and BMI percentile values are presented as a mean and SD, and all other values are sample size and percent of the total sample. Afterschool weather varied across all three data collection periods. During baseline (fall), the average afterschool temperature was 19.3 °C and sunset occurred at 5:44 PM. In winter, following the first intervention period (post I), the average afterschool temperature was −6.2 °C and sunset occurred at 4:17 PM. At the end of the second intervention period in spring (post II), the average afterschool temperature was 10.0 °C and sunset occurred at 6:33 PM. Throughout all three data collection periods, no measurable precipitation occurred, relative outdoor humidity averaged between 60–70%, and average wind speed ranged from 15.6–20.6 km/h.

### 3.2. Afterschool Physical Activity and Sedentary Behaviors

Of the total participants that consented to participate in this study from grades 3 (*n* = 22), 4 (*n* = 36), and 6 (*n* = 41), some participant attrition occurred at each measurement period. Among participants from third grade, data from 20 participants were used at baseline, and data from 19 participants was used during post I and post II, respectively. Among participants from fourth grade, 34 participants provided data for analysis at baseline, followed by 29 at post I, and 31 at post II. Finally, among participants from sixth grade, 40 participants provided data for analysis at baseline, followed by 35 at post I, and 38 at post II.

There were no significant differences in afterschool PA behaviors between Group 1 (sit–stand) and Group 2 (stand–sit) at baseline and following both intervention periods (post I and post II; Table 3). Participants spent the greatest proportion of afterschool time engaging in LPA and MVPA at baseline, with both variables decreasing, and SB increasing, at each subsequent assessment period.

Caregiver-reported sports and extracurricular participation (Table 3) revealed that the majority of students in the study were engaged in at least one extracurricular afterschool activity at baseline, post I, and post II. Although sport participation was lower in the winter (post I) compared to baseline and the spring (post II), there were no significant differences between groups across all three measurement periods.

At baseline, the majority of participants (59.79%) engaged in active transportation home from school every day of the week. During the post I assessment (winter), there was a significant difference between groups in the number of participants engaging in active transportation home from school, with a greater proportion of Group 1 (63.89%) participating on all days of the week, whereas a greater proportion of Group 2 (25.53%) reported no days engaging in active transportation home from school (*p* = 0.0488). This trend of more participants from Group 1 engaging in active transportation home from school compared to Group 2 continued at post II (spring); however, differences were not statistically significant.

### 3.3. Examination of In-School and Afterschool Moderate- to Vigorous-Intensity Physical Activity Levels

Table 4 presents the total number of participants who did or did not accumulate at least 30 min of MVPA in school and after school at baseline, post I, and post II. The distribution of participants meeting or not meeting the threshold of 30 min of MVPA in school or after school was not significantly different within the baseline (*p* = 0.63) and post II (*p* = 0.13) measurement periods. This was in contrast with the post I measurement period, where a significantly greater number of participants did not accumulate 30 min or more of MVPA both in school and after school, compared to those meeting the 30-min threshold in school or after school (*p* < 0.01). Additionally, during the post I measurement period, participants were 0.35 times as likely to attain 30 or more minutes of MVPA after school compared to the in-school period.

### 3.4. Interaction Effects between Desk-Assignment, After-School Physical Activity, and Sedentary Behavior

Results from a mixed-effects model using Group 1 as a reference found no significant two- or three-way interactions between desk assignment (standing or sitting), time, and participants afterschool SB (estimate [minutes] ± SE; *p* = 0.54), LPA, (*p* = 0.44), or MVPA (*p* = 0.96) (Table 5). However, significant afterschool differences in SB and MVPA were found between sexes, with boys spending less time in SB (−3.43 ± 1.53 min; *p* = 0.03), and more time in MVPA (0.31 ± 0.09 min; *p* < 0.01). Significant differences were also found across grade levels, with sixth-grade students spending more afterschool time in SB (5.49 ± 2.02 min; *p* < 0.01) and less time in LPA (−4.13 ± 1.51 min; *p* < 0.01). When comparing intervention periods to baseline across the school year, it was found that all participants, regardless of desk assignment, engaged in significantly more SB when measured at post I (3.61 ± 1.13 min; *p* < 0.01) and post II (3.34 ± 1.11 min; *p* < 0.01), compared to baseline. Finally, in-school PA and SB had a significant association with children’s afterschool behaviors, with more SB in school associated with more afterschool SB (0.19 ± 0.07 min; *p* = 0.01), more LPA in school associated with more LPA after school (0.21 ± 0.08 min; *p* < 0.01), and more MVPA in school associated with more MVPA after school (0.29 ± 0.07 min; *p* < 0.01).

## 4. Discussion

The results of this analysis suggest that desk assignment during the school day had little impact on elementary student’s afterschool SB, LPA, or MVPA. Although participants experienced changes in afterschool activity behaviors throughout the school year, students assigned to a stand-biased desk in the classroom did not significantly alter behaviors during the afterschool period relative to those assigned to a traditional seated desk across both intervention periods.

Throughout all three data collection periods, no significant differences in afterschool PA or SB were recorded between stand-biased or traditional desk groups, with similar trends observed across all participants as the school year progressed. Participants were most active at the beginning of the school year, spending an average of 9% of afterschool wear time in MVPA, 30% in LPA, and 62% in SB. During the two subsequent measurement periods occurring in winter and spring (post I and post II), participants increased the proportion of afterschool wear time they spent in SB (post I: 65.6%; post II: 65.7%), whereas both MVPA (post I: 6.8%; post II: 7.4%) and LPA (post I: 27.6%; post II: 26.8%) declined proportionately. Although the decrease in PA levels from fall to winter is consistent with previous literature [38,39,40,41], participants’ activity levels did not rebound substantially from winter to spring, remaining lower than baseline. Throughout the school year, seasonal variation in children’s daily activity behaviors suggests that PA levels are at their highest at the beginning of the school year in the fall, and decrease into the winter period before rebounding in the spring as summer approaches [38]. The afterschool period may be influenced by caregiver oversight or seasonal sport participation which guides opportunities to engage in PA and SB. However, others may have free time where they have the option to choose whether to engage in PA. Overall, variations in geographical location and acute weather events can present greater barriers to activity during the afterschool period than during the school day and have a larger impact on outdoor play spaces [42,43]. In the current study, the shorter and colder days of winter may have contributed to participants being significantly less likely to accumulate more than 30 min of MVPA after school compared to the in-school period. These findings lend support to the need for fostering an environment that allows for greater amounts of PA in school during times when the likelihood of obtaining sufficient PA after school is significantly reduced. Furthermore, the time at which the post II measurement period took place might be considered “early spring”. During this time, access to outdoor spaces for activity may be limited due to the ground being saturated by water. Additionally, many outdoor sport seasons had yet to begin when post II assessments took place, potentially limiting opportunities for PA after school. To support children’s engagement in PA after school, it is important that opportunities are provided, regardless of environmental obstacles which otherwise encourage increases in SB.

Afterschool sport participation may be a large contributor to children’s PA levels; however, it has been suggested that children who participate in sports are likely to already be more active than their peers [44]. In the present analysis, 83% of respondents reported that their child participated in sports at baseline according to a caregiver survey, coinciding with the most active afterschool measurement period. Subsequent survey responses revealed that the proportion of children participating in afterschool sports during the winter decreased to 62%, before increasing once more to a participation rate of 84% in the spring. In this elementary school, afterschool sports participation is typically affiliated with an organization or club rather than a school, potentially limiting student access. In a review published by Somerset and Hoare [45], the primary perceived barriers to after-school sport participation included time and cost, although friendships, accessibility, skill/ability, and fear of judgement have also been reported. In the present analysis, seasonal variation in sport participation may partially explain the trend in PA and SB from the fall to winter periods, yet the rebound in sports participation from winter to spring did not coincide with a similar rebound in accelerometer-recorded PA or SB. Without further information however, it can only be speculated that a proportion of children engaging in afterschool sports removed their accelerometer devices during this time out of necessity (i.e., water sports) or for improved comfort (i.e., contact sports); therefore only recording less-structured times during the afterschool period. The association between caregiver-reported sport participation and activity levels warrants further investigation.

Research examining the afterschool period suggests that children accumulate a significant proportion of their recommended daily PA during this time. Specifically, published research has shown that children accumulate 29–33% (18–20 min) of the recommended daily PA during the afterschool period [46,47]. Similar to published literature, the present analysis showed that elementary school children accumulated approximately one-third of the recommended daily MVPA after school. This time spent in MVPA after school was consistent across all three measurement periods (fall, winter, spring) irrespective of classroom desk assignment. Although the afterschool period is shorter than the time children spend in school, this segment of the day can significantly contribute to total daily PA levels among youth. Moreover, data from the current analysis suggests that children’s afterschool PA behaviors may be resistant to change following interventions targeting sedentary behaviors in the classroom; however further research is necessary to understand the full impact of school-based interventions on total daily PA.

This secondary analysis was not without its limitations. Due to the need to align with the academic calendar, the final measurement period (post II) only assessed three afterschool days instead of the four assessed during baseline and post I. To account for this, proportions of weekly afterschool wear time were used to analyze data, as this was deemed more representative of normal afterschool behavior than presenting activity levels in daily minutes. It is also acknowledged that the inclusion of participants with just one valid day of accelerometer data has the potential to add bias to results. Analysis of the data excluding these participants did not significantly alter outcomes; therefore a minimum criterion of one valid day was maintained. The response rate on the caregiver surveys was low and many accelerometer wear logs were incomplete. Together, this missing information made it difficult to determine whether afterschool non-wear time was attributable to a period of organized sports or simply not wearing the device. Again, to address this limitation, participants’ SB, LPA, and MVPA are presented as a proportion of afterschool wear time only, excluding periods of non-wear.

At the same time, however, this analysis contained multiple strengths. First, by implementing a within-classroom design where only half of all desks in each participating classroom were exchanged for stand-biased desks, this study was able to capture the impact of altering a classroom environment on afterschool activities, while minimizing the impact which a different teacher might have on students’ behaviors. Furthermore, although only students who consented to participate in this study provided usable data, all students in participating classrooms, regardless of enrollment in the study, were given the opportunity to use either a traditional or stand-biased desk alongside their peers, supporting an inclusive classroom environment. Although precipitation and wind speed have been shown to be negatively associated with children’s PA levels [48], the data collection periods for this analysis experienced negligible amounts of precipitation and minimal variation in wind speed, reducing the impact of weather. Finally, this study employed the use of objective monitors of PA and SB in the form of waist-worn accelerometers, which quantify directional acceleration into time spent active (PA) or inactive (SB). In contrast to self- and proxy-report surveys or other subjective assessments of PA and SB which are limited by question interpretability, participant recall, and social desirability bias [49], accelerometers present a valid measure of total activity, capable of better capturing the brief, sporadic bouts of PA which children are known to engage in [39], as well as the patterns and intensities in which these activities occur [50,51,52].

## 5. Conclusions

In conclusion, the use of stand-biased desks in elementary school classrooms did not have a detrimental impact on the afterschool PA and SB of elementary school children. The afterschool period makes up a large portion of the total day, and has the ability to provide children with opportunities to accumulate a significant proportion of PA. With the knowledge that altering the classroom environment to influence students PA and SB in school does not negatively impact activity levels across other periods of the day, researchers, administrators, teachers, and caregivers should continue exploring ways in which children can be encouraged to engage in greater levels of PA during and after the school day.

## Figures and Tables

**Table 1:** Valid accelerometer afterschool wear days.

		Number of Valid Days *					
Measurement Period		0	1	2	3	4	Total
Baseline (Fall)	Freq.	8	8	17	21	45	99
	(%)	(8.1)	(8.1)	(17.2)	(21.2)	(45.5)	
Post I (Winter)	Freq.	21	12	13	23	27	96
	(%)	(21.9)	(12.5)	(13.5)	(24)	(28.1)	
Post II (Spring)	Freq.	18	10	20	47	N/A	95
	(%)	(19)	(10.5)	(21.1)	(49.5)		
Total		47	30	50	91	72	290

Note: Participants were included in analysis when providing one or more days of valid afterschool wear time. * Valid wear day: ≥3 h 47 min of wear time following school release. N/A, post II assessment occurred during a time where just three days of after-school wear time were able to be collected.

**Table 2:** Participant characteristics.

	Total Sample		Grade 3		Grade 4		Grade 6		
	(*n* = 99, 100%)		(*n* = 22, 22.2%)		(*n* = 36, 36.4%)		(*n* = 41, 41.4%)		
Characteristics	Mean or *n*	SD or %	Mean or *n*	SD or %	Mean or *n*	SD or %	Mean or *n*	SD or %	*p*
Age (Year)	10.2	1.4	8.5	0.5	9.6	0.6	11.7	0.5	<0.0001 ^a^
Height (cm)	146.0	29.3	134.8	7.3	146.3	46.5	152.0	7.4	<0.0001 ^a^
Body Mass (kg)	37.0	9.9	29.3	4.3	34.1	8.9	43.8	8.7	<0.0001 ^a^
BMI Percentile	52.6	29.9	53.0	25.5	51.0	29.0	53.9	33.3	0.9180 ^a^
Male	56	57.1	15	68.18	15	41.67	26	65.0	0.0601 ^b^
Race									
White	69	79.3	18	90	21	67.7	30	83.3	0.3264 ^c^
Black/African American	3	3.5	1	5.0	2	6.5	0	0.0	
Asian	8	9.2	0	0.0	4	12.9	4	11.1	
Mixed Race	7	8.1	1	5.0	4	12.9	2	5.6	
Hispanic	8	9.4	2	10	1	3.23	5	14.7	0.4340 ^c^
Family Income									
Under $49,999	12	14.0	0	0.0	7	23.3	5	14.3	0.3307 ^c^
$50,000–$99,999	20	23.3	5	23.8	8	26.7	7	20.0	
$100,000–$149,999	23	26.7	6	28.6	5	16.7	12	34.3	
$150,000–$199,999	10	11.6	3	14.3	4	13.3	3	8.6	
$200,000 or more	21	24.4	7	33.3	6	20.0	8	22.9	

Note: *p*-values indicating differences between grade levels obtained by using Kruskal–Wallis ^a^, chi-square ^b^, and Fisher’s exact tests ^c^.

**Table 3:** Physical activity and sedentary behavior characteristics afterschool in those using a sitting desk versus a stand-biased desk (mean (SD)).

	Baseline (Fall)					Post I (Winter)					Post II (Spring)				
	Group 1 Sit-Stand		Group 2 Stand-Sit			Group 1 Sit-Stand		Group 2 Stand-Sit			Group 1 Sit-Stand		Group 2 Stand-Sit		
	(*n* = 39)		(*n* = 58)			(*n* = 36)		(*n* = 47)			(*n* = 37)		(*n* = 52)		
Afterschool Activity	MEAN	SD	MEAN	SD	P_1_ ^a^	MEAN	SD	MEAN	SD	P_2_ ^a^	MEAN	SD	MEAN	SD	P_3_ ^a^
SB (%)	60.0	8.3	62.4	9.8	0.2256	64.7	8.3	66.2	10.5	0.4469	64.7	9.7	66.5	10.2	0.4219
LPA (%)	30.7	5.7	29.5	7.3	0.4364	28.3	6.0	27.1	8.3	0.4066	27.8	7.8	26.1	7.6	0.3006
MVPA (%)	9.4	4.4	8.1	4.2	0.2213	7.0	4.2	6.7	3.7	0.8880	7.5	3.6	7.4	4.6	0.5669
Average Daily Wear Time (h/day)	11.3	1.7	10.9	2.9	0.2605	11.3	2.7	10.4	3.6	0.4079	10.9	2.4	10.7	3.2	0.9868
Average Afterschool Wear Time (h/day)	5.4	0.5	5.4	0.4	0.8151	5.4	0.5	5.4	0.5	0.4543	5.5	0.5	5.4	0.5	0.5203
Afterschool Wear Time (Total Hours)	19.9	5.7	18.6	6.6	0.2334	17.7	7.4	16.9	8.2	0.8850	14.4	6.0	13.6	5.2	0.3379
	**N**	**%**	**N**	**%**	**P_1_**	**N**	**%**	**N**	**%**	**P_2_**	***n***	**%**	***n***	**%**	**P_3_**
Sport Participation (**Yes**/No)—Caregiver Survey	33	83.9	36	81.8	0.8171 ^b^	15	68.2	18	58.1	0.4540 ^b^	18	85.7	23	82.1	1.0000 ^b^
Active Transport Home from School															
0 Days	4	10.5	8	14.3	0.4379 ^c^	3	8.3	12	25.5	0.1868 ^c^	4	11.1	14	28.6	0.0525 ^c^
1 Day	2	5.3	2	3.6		4	11.1	5	10.6		3	8.3	3	6.1	
2 Day	3	7.9	11	19.6		3	8.3	4	8.5		2	5.6	4	8.2	
3 Day	1	2.6	5	8.9		3	8.3	5	10.6		5	13.9	6	12.2	
4–5 Days	28	73.7	30	53.6		23	63.9	21	44.7		22	61.1	22	44.9	
Treatment Condition (Sit/Stand)	Sit		Sit			Sit		Stand			Stand		Sit		

Note: P_1_, test between groups at baseline; P_2_, test between groups at post I; P_3_, test between groups at post II. *p*-values obtained from independent sample *t*-tests ^a^, chi-square tests ^b^, and Wilcoxon rank sum tests ^c^.

**Table 4:** Distribution of moderate- to vigorous-intensity physical activity during the in-school and afterschool periods.

		Baseline (Fall)				Post I (Winter)				Post II (Spring)			
		Afterschool PA (>30 min MVPA)				Afterschool PA (>30 min MVPA)				Afterschool PA (>30 min MVPA)			
In-School PA (>30 min MVPA)		NO	YES	TOTAL	*p*	NO	YES	TOTAL	*p*	NO	YES	TOTAL	*p*
NO	N	26	18	44	0.6310	40	8	48	0.0071	33	10	43	0.1306
	%	59.1	40.9			83.3	16.7			76.7	23.3		
YES	N	21	24	45		23	4	27		18	16	34	
	%	46.7	53.3			85.2	14.8			52.9	47.1		
TOTAL	N	47	42	89		63	12	75		51	26	77	
	Odds Ratio	0.86				0.35				0.56			
	Lower CI	0.46				0.16				0.26			
	Upper CI	1.61				0.78				1.20			

Note: *p*-values obtained using McNemar’s test. Odds ratio, odds of attaining >30 min of MVPA after school compared to in school; CI, 95% confidence interval.

**Table 5:** Results from a mixed-effects model assessing interactions between sex, grade, desk assignment, time, and in-school activity on after-school SB, LPA, and MVPA.

	Sedentary Behavior (SB)			Light PA (LPA)			Moderate–Vigorous PA (MVPA)		
Effects	EST	SE	*p*	EST	SE	*p*	EST	SE	*p*
Intercept	48.87	4.49	<0.0001	25.47	3.16	<0.0001	1.33	0.19	<0.0001
Male	−3.43	1.53	0.0263	0.87	1.15	0.4474	0.31	0.09	0.0005
Grade (REF: 3rd Grade)			0.0264			0.0257			0.3472
4th Grade	2.96	1.98	0.1379	−2.56	1.50	0.0891	−0.02	0.11	0.8565
6th Grade	5.49	2.02	0.0074	−4.13	1.51	0.0069	−0.14	0.11	0.2192
Group (REF: Group 1)	0.89	1.44	0.5373	−0.84	1.09	0.4424	0.00	0.08	0.9545
Time (REF: Baseline)			0.0020			0.0067			0.0163
Post I	3.61	1.13	0.0016	−2.13	0.86	0.0144	−0.19	0.06	0.0042
Post II	3.34	1.11	0.0029	−2.55	0.85	0.0031	−0.09	0.06	0.1437
School Time Activity (SB, LPA, MVPA)	0.19	0.07	0.0133	0.21	0.08	0.0069	0.29	0.07	0.0001

Note: No significant interaction (both 2-way and 3-way) were found between desk type and physical activity or sedentary behaviors. REF, reference group used for model comparisons. School time activity is a reference of the in-school levels of the same behavior being compared (i.e., in-school SB to afterschool SB; in-school LPA to afterschool LPA; in-school MVPA to afterschool MVPA).

## Data Availability

Not applicable.
